# Effect and mechanism of Nintedanib on acute and chronic radiation-induced lung injury in mice

**DOI:** 10.1371/journal.pone.0324339

**Published:** 2025-05-23

**Authors:** Kun Zhang, Lu Ren, Yujie Zhai

**Affiliations:** 1 Department of Oncology, Binzhou Medical University Hospital, Binzhou, Shandong, P.R.China; 2 Department of Oncology, Jieshou City People’s Hospital, Jieshou Hospital Affiliated to Anhui Medical College, Jieshou, Anhui, P.R.China; 3 Department of Hematology, Jieshou City People’s Hospital, Jieshou Hospital Affiliated to Anhui Medical College, Jieshou, Anhui, P.R.China; Fujian Provincial Hospital, CHINA

## Abstract

**Objective:**

To investigate the efficacy of Nintedanib in treating acute and chronic radiation-induced lung injury and its mechanism of action.

**Methods:**

A radiation-induced lung injury model was established in mice using 6MV X-rays at 18Gy to irradiate the lungs. The mice were randomly divided into four groups: control group, radiation therapy group, low-dosage Nintedanib + radiation therapy group, and high dosage Nintedanib + radiation therapy group. The mice were euthanized on day 14 and 3 months post-radiation to observe changes in acute and chronic inflammation and the expression of related proteins.

**Results:**

Compared to the radiation therapy group, the low and high-dosage Nintedanib groups showed varying degrees of improvement in mental state, responsiveness, food and water intake, and fur condition. During the acute inflammatory phase, HE staining revealed inflammatory changes in the lung tissues of both Nintedanib groups, but the pathology was less severe than in the radiation group, with the high-dosage group showing more significant reduction. Serum levels of IL-6, TNF-α and TGF-β1 were significantly reduced (P < 0.05), suggesting that Nintedanib can decrease the expression of serum inflammatory factors. The percentage of Smad2-positive area in the low and high-dosage Nintedanib groups was (7.395 ± 0.90)% and (5.577 ± 1.56)%, respectively, both significantly lower than the radiation group (P < 0.05). At 3 months post-radiation, Masson’s trichrome staining showed that the Ashcroft score in the Nintedanib groups was significantly lower than in the radiation group (P < 0.05). There were statistically significant differences between the low and high-dosage groups in the percentage of Smad2 and αSMA-positive areas and the levels of serum TGF-β1 (all P < 0.05), and both were significantly lower compared to the radiation group (P < 0.05).

**Conclusion:**

(1) Nintedanib can improve the general condition of mice with acute and chronic radiation-induced lung injury and reduce pathological damage to lung tissue. (2) Nintedanib may exert a protective effect on mice with acute and chronic radiation-induced lung injury by downregulating the TGF-β1/Smad2 signaling pathway, thereby inhibiting inflammatory and fibrotic responses.

Radiation therapy remains a cornerstone in managing early-stage inoperable lung cancer and locally advanced disease [1]. However, radiation-induced lung injury (RILI), encompassing acute radiation pneumonitis (RP) and chronic radiation-induced pulmonary fibrosis (RILF), poses a significant clinical challenge, affecting 15–30% of patients undergoing thoracic radiotherapy [2]. The biphasic pathology of RILI involves early inflammatory responses within 3–4 months post-radiation [3], characterized by cytokine storms and immune cell infiltration, followed by progressive fibrosis marked by fibroblast activation and extracellular matrix (ECM) deposition [4–6]. These processes not only compromise pulmonary function but frequently necessitate treatment interruptions, ultimately diminishing oncological outcomes [7].

Current management strategies for RILI remain suboptimal. While glucocorticoids transiently suppress acute inflammation, they lack efficacy against fibrotic progression and carry risks of immunosuppression and metabolic complications [8,9]. Moreover, no FDA-approved therapies directly target the molecular drivers of radiation fibrosis, highlighting a critical unmet need [10]. This therapeutic gap underscores the urgency to identify agents capable of simultaneously modulating inflammatory and fibrotic pathways – a dual mechanism essential for addressing RILI’s biphasic nature.

Nintedanib, a multi-target tyrosine kinase inhibitor approved for idiopathic pulmonary fibrosis (IPF) and progressive fibrosing interstitial lung diseases, presents a compelling candidate [11–13]. Its triple angiokinase inhibition (targeting VEGFR, PDGFR, and FGFR) intersects with key pathways implicated in both radiation-induced inflammation and fibrosis [14]. Preclinical evidence suggests Nintedanib attenuates TGF-β1 signaling – the master regulator of fibrogenesis – by blocking Smad2/3 phosphorylation and downstream ECM remodeling [15–17]. Notably, TGF-β1 overexpression persists for weeks post-radiation, driving fibroblast-to-myofibroblast transition and epithelial-mesenchymal crosstalk [18,19], processes potentially modifiable by Nintedanib. Furthermore, its anti-inflammatory properties, including suppression of TNF-α/IL-6 cascades and NF-κB activation [20,21], may disrupt the feedforward loop between early inflammation and late fibrosis.

Despite these mechanistic rationales, Nintedanib’s therapeutic potential in RILI remains underexplored, particularly its capacity to concurrently mitigate acute and chronic injury phases. This study investigates Nintedanib’s efficacy in a murine RILI model, focusing on its dual modulation of inflammatory cytokines and fibrotic signaling through the TGF-β1/Smad2 axis. Our findings aim to bridge the critical gap between IPF-directed therapies and radiation-specific lung injury management, offering a translational framework for combinatorial approaches in oncological care.

## Materials and methods

1. Experimental animals and grouping

Eighty male C57BL/6 mice (8 weeks old, 20–25 g) were purchased from Jinan Pengyue Experimental Animal Breeding and housed under controlled temperature (22 ± 1°C) and humidity (55 ± 5%) with a 12-hour light-dark cycle. After a 7-day acclimatization period, mice were randomly assigned using a computer-generated randomization table to four groups (n = 20/group): (1) Control group (saline gavage), (2) Radiation therapy (RT) group, (3)Low-dosage Nintedanib(40 mg/kg) + radiation therapy group, and (4)high dosage Nintedanib(80 mg/kg)+ radiation therapy group. Body weight was monitored every three days. Mice losing ≥20% body weight within 24 hours were euthanized via cervical dislocation under anesthesia (sodium pentobarbital, 50 mg/kg). All procedures were approved by the Animal Ethics Committee of Binzhou Medical University Hospital (No. 20230206–54) and complied with ARRIVE guidelines.

2. Preparation of acute Radiation-Induced Lung Injury(RILI) model

Mice were anesthetized via intraperitoneal injection of 0.3% sodium pentobarbital (50 mg/kg) and placed supine on a thermoplastic immobilization device. The thoracic region was exposed using a custom lead collimator (2 × 3 cm field), and a single dose of 18 Gy was delivered using a 6-MV linear accelerator (Varian Medical Systems) at a dose rate of 300 MU/min, source-to-surface distance (SSD) 100 cm. Anesthesia depth was confirmed by absence of pedal reflex. Sham-irradiated controls underwent identical procedures without beam activation.

3. Drug administration and sampling

Once the mice fully recovered, the control and radiation groups received a gavage of physiological saline at a dose of 0.01 ml/g. The low-dosage group was administered nidanimib at 40 mg/kg, while the high-dosage group received nidanimib at 80 mg/kg, with all groups being treated once daily. Ten mice from each group were euthanized on day 14 and again at three months post-irradiation. The mice were anesthetized via intraperitoneal injection of 0.3% sodium pentobarbital at a dose of 50 mg/kg. Approximately 0.6 to 0.8 milliliters of blood were collected from each mouse’s eye and then incubated in a centrifuge tube at room temperature for more than two hours. Following this, cervical dislocation was performed to euthanize the mice, after which the lungs were removed. Fresh lung tissue from the mice was also collected; excess trachea was trimmed off, rinsed with physiological saline, surface water was absorbed with filter paper, and the tissue was fixed with 10% formaldehyde.

4. Histopathological and immunohistochemical analysis

H&E and Masson’s trichrome staining were performed using commercial kits. Immunohistochemistry for Smad2 and αSMA included antigen retrieval and DAB visualization. Slides were digitized and the results were evaluated. The Szapiel score was used to evaluate pulmonary fibrosis stained with H&E [22]. Pulmonary fibrosis stained with Masson trichrome was evaluated according to the Ashcroft score [23].

5. Enzyme-linked Immunosorbent Assay (ELISA)

We measured the serum concentrations of interleukin-6 (IL-6), tumor necrosis factor-alpha (TNF-α), and transforming growth factor-beta1 (TGF-β1) using ELISA kits from Boster Biological Technology co.ltd, ensuring that all operational steps were strictly adhered to as per the provided instructions.

6. Statistical methods

We analyzed the experimental data using GraphPad Prism 10.0 software, expressing quantitative data as mean ± standard deviation (X ± S). We conducted comparisons among multiple groups using analysis of variance and performed pairwise comparisons with the Tukey test, considering a P-value of less than 0.05 as statistically significant.

### Results

1. Nintedanib enhances survival and alleviates radiation-induced weight loss in mice

**Fig 1:**
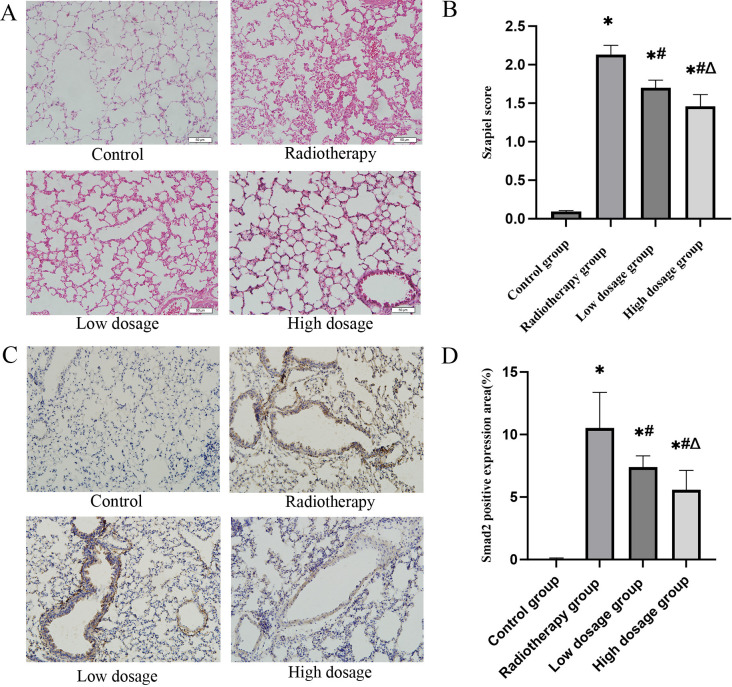
Therapeutic effects of Nintedanib on acute radiation-induced lung injury in mice. (A) Pathological changes in lung tissues of mice in each group (HE staining 200×). (B) Lung tissue szapiel score of mice in each group(Compared with the control group, *P ＜ 0.05; Compared with the radiotherapy group, # P ＜ 0.05; Compared with the low dosage group, △P ＜ 0.05).(C)Immunohistochemical staining for the expression of Smad2 in lung tissues(200×).(D) The percentage of Smad2 positive expression area (Compared with the control group, *P ＜ 0.05; Compared with the radiotherapy group, # P ＜ 0.05; Compared with the low dosage group, △P ＜ 0.05).

During the acute phase (days 1–14), irradiated mice (RT group) exhibited lethargy, reduced activity (62% decrease in wheel-running vs. control, P < 0.01; Fig 1A), and diminished food intake (2.1 ± 0.3 g/day vs. 4.5 ± 0.4 g in controls, P < 0.001). Nintedanib treatment dose-dependently improved these parameters, with the high-dosage group showing near-normal activity levels (3.8 ± 0.5 g/day food intake, P < 0.05 vs. RT group).

**Fig 2:**
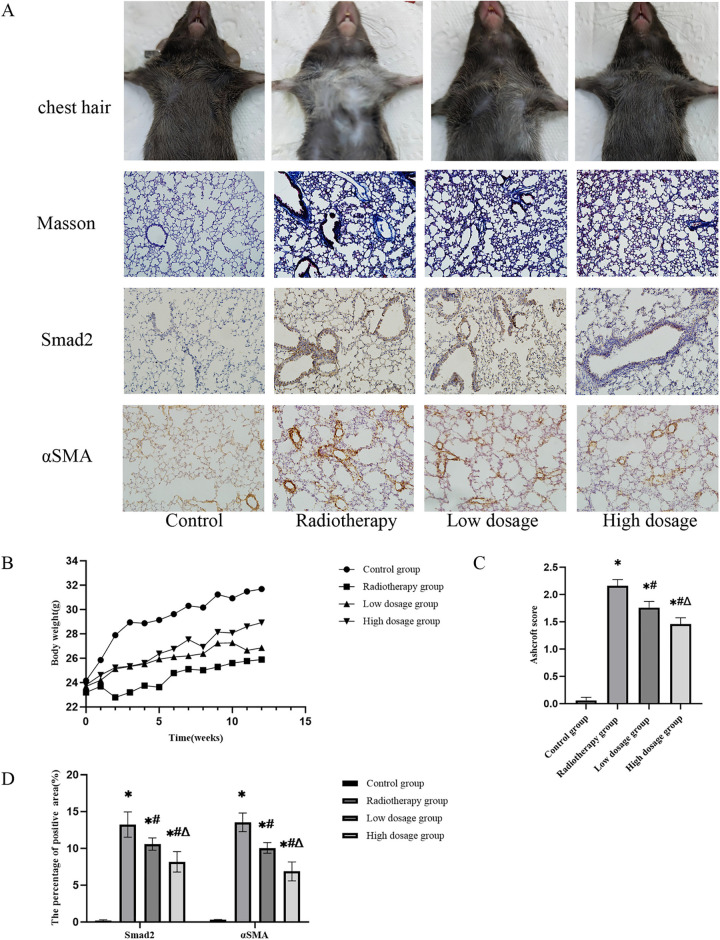
Long-term protective effects of Nintedanib on chronic radiation-induced lung injury in mice. (A) The chest hair condition of mice in each group at 3 months after radiotherapy;Pathological changes in lung tissues of mice in each group (Masson staining 200×);Immunohistochemical staining for the expression of Smad2 and αSMA in lung tissues(200×).(B) The change trends of mice body weight. (C) Lung tissue ashcroft score of mice in each group(Compared with the control group, *P ＜ 0.05; Compared with the radiotherapy group, # P ＜ 0.05; Compared with the low dosage group, △P ＜ 0.05). (D) The percentage of Smad2 and αSMA positive expression area(Compared with the control group, *P ＜ 0.05; Compared with the radiotherapy group, #P ＜ 0.05; Compared with the low dosage group, △P ＜ 0.05).

By 3 months post-irradiation, RT group mice displayed persistent weight loss (25.65 ± 0.75 g vs. control 31.87 ± 1.87 g, P < 0.05) and graying of fur (Fig 2A). Nintedanib attenuated these effects, with the high-dosage group achieving significantly higher body weight (28.21 ± 1.11 g, P < 0.05 vs. RT group; Fig 2B).

2. Nintedanib suppresses radiation-induced acute inflammation and TGF-β1-driven fibrotic signaling

At 14 days post-RT, serum levels of IL-6 (53.33 ± 5.338 pg/mL vs. control 9.957 ± 2.850 pg/mL, P < 0.001) and TNF-α (57.47 ± 2.932 pg/mL vs. 24.39 ± 3.131 pg/mL, P < 0.001) were markedly elevated in the RT group. Nintedanib dose-dependently reduced these cytokines (IL-6: 32.76 ± 3.654 pg/mL in the low-dosage group and 24.90 ± 3.547 pg/mL in the high-dosage group; TNF-α: 46.25 ± 3.321 pg/mL and 35.41 ± 2.931 pg/mL, respectively; P < 0.05 vs. RT group; Table 1).

**Table 1:** Level of IL-6, TNF-α and TGF-β1 in the serum of mice in each group (‾*X* ± *S*, pg/mL, N = 10).

Group	IL-6	TNF-α	TGF-β1
Control group	9.957 ± 2.850	24.39 ± 3.131	2069.62 ± 100.7
Radiotherapy group	53.33 ± 5.338	57.47 ± 2.932	14966.9 ± 552.9
Low dosage group	32.76 ± 3.654	46.25 ± 3.321	7462.11 ± 370.2
High dosage group	24.90 ± 3.547	35.41 ± 2.931	6732.34 ± 265.9

Concurrently, TGF-β1 levels increased sharply post-RT (RT group: 14,966.9 ± 552.9 pg/mL vs. control 2,069.62 ± 100.7 pg/mL, P < 0.001; Table 1), with sustained elevation at 3 months (RT group: 17,626.6 ± 759.3 pg/mL vs. control 2,097.56 ± 263.2 pg/mL, P < 0.001; Table 2). Nintedanib significantly attenuated TGF-β1 secretion (3-month levels: low-dosage 10,820.0 ± 874.4 pg/mL, high-dosage 8,923.88 ± 625.1 pg/mL; P < 0.05 vs. RT group; Table 2), highlighting its dual role in mitigating both acute inflammation and chronic fibrotic signaling.

**Table 2:** Level of TGF-β1 in the serum of mice in each group (‾*X* ± *S*, pg/mL, N = 10).

Group	TGF-β1
Control Group	2097.56 ± 263.2
Radiotherapy Group	17626.6 ± 759.3
Low Dosage Group	10820.0 ± 874.4
High Dosage Group	8923.88 ± 625.1

3. Nintedanib suppresses acute and chronic Smad2 activation and attenuates myofibroblast differentiation via αSMA downregulation in a time- and dose-dependent manner

Immunohistochemical analysis revealed that radiotherapy markedly increased Smad2 expression in lung tissue, with the radiotherapy group showing a 10.52 ± 2.85% positive area at 14 days post-irradiation, significantly higher than controls (0.069 ± 0.06%, P < 0.05). Nintedanib treatment dose-dependently reduced Smad2 levels to 7.395 ± 0.90% (low-dose) and 5.577 ± 1.56% (high-dose) (P < 0.05 vs. RT group; Fig 1C, D). By 3 months post-irradiation, Smad2 expression further escalated in the RT group (13.96 ± 1.83%), while both Nintedanib doses maintained significant suppression (low-dose: 10.59 ± 0.83%; high-dose: 8.177 ± 1.39%; P < 0.05), demonstrating persistent pathway inhibition.

Radiation-induced fibrotic progression was further evidenced by elevated αSMA expression, a marker of myofibroblast differentiation, in the RT group (13.54 ± 1.26% at 3 months). Nintedanib dose-dependently suppressed αSMA levels, with the high-dose group achieving a greater reduction (6.876 ± 1.29%) compared to the low-dose group (10.06 ± 0.73%) (P < 0.05; Fig 2A, D). These findings collectively indicate that Nintedanib inhibits TGF-β1/Smad signaling in a time- and dose-dependent manner, effectively disrupting both acute Smad2 activation and chronic αSMA-driven fibrotic remodeling.

4. Nintedanib attenuates radiation-induced lung injury across acute and chronic phases

Acute Phase (14 days): H&E staining demonstrated severe alveolar wall thickening (Szapiel score: RT group 8.2 ± 1.1 vs. control 1.0 ± 0.3, P < 0.001) and inflammatory infiltration in RT mice, which were ameliorated by Nintedanib (low-dosage: 5.4 ± 0.8, high-dosage: 3.1 ± 0.6; P < 0.05; Fig 1B).

Chronic Phase (3 months): Masson’s trichrome staining revealed extensive collagen deposition in RT mice (32.5 ± 4.7% blue area vs. control 2.1 ± 0.8%, P < 0.001), corresponding to elevated Ashcroft scores (RT group: 6.8 ± 0.9 vs. control 0.5 ± 0.2, P < 0.001). Nintedanib reduced fibrosis in a dose-dependent manner (low-dosage: 18.4 ± 3.2%, Ashcroft 4.2 ± 0.7; high-dosage: 12.6 ± 2.1%, Ashcroft 2.9 ± 0.5; P < 0.01 vs. RT group; Fig 2C).

## Discussion

Radiation-induced lung injury (RILI), comprising acute pneumonitis (RP) and chronic fibrosis (RILF), involves a dynamic interplay of inflammatory and fibrotic pathways. Our study demonstrates that Nintedanib, a multi-kinase inhibitor, effectively mitigates both acute and chronic phases of RILI in a murine model, potentially through suppression of the TGF-β1/Smad2 signaling axis. These findings align with its established role in idiopathic pulmonary fibrosis (IPF) and progressive fibrosing interstitial lung diseases [8,9,24], suggesting shared pathogenic mechanisms across fibrotic disorders.

In acute RILI, Nintedanib reduced serum TNF-α and IL-6 levels, key cytokines driving radiation-induced inflammation [10,11,25]. This anti-inflammatory effect likely disrupts the NF-κB-mediated amplification of immune cell recruitment and oxidative stress [26–29], critical contributors to early tissue damage. Notably, the dose-dependent attenuation of these cytokines underscores Nintedanib’s capacity to modulate the pro-inflammatory milieu, consistent with its reported inhibition of TNF-α-driven IL-6 cascades [30]. During the chronic phase, Nintedanib suppressed α-SMA expression and collagen deposition, markers of fibroblast-to-myofibroblast differentiation and ECM accumulation [14,31]. This dual activity—targeting both inflammation and fibrosis—positions Nintedanib as a multifaceted therapeutic agent for RILI, addressing its biphasic pathology.

Central to these effects is Nintedanib’s interaction with the TGF-β1/Smad2 pathway, a master regulator of fibrogenesis [7,32–34]. Radiation triggers persistent TGF-β1 elevation, peaking at 2–4 weeks post-exposure [13], which activates Smad2/3 to drive fibroblast proliferation, EMT, and ECM remodeling [35,36]. Our data reveal that Nintedanib reduces TGF-β1 levels and Smad2 activation, mirroring its inhibition of receptor tyrosine kinases (e.g., PDGFR, FGFR) implicated in TGF-β1 signaling crosstalk [37–40]. By blocking these upstream kinases, Nintedanib may prevent Smad2 phosphorylation and nuclear translocation, thereby interrupting transcription of fibrotic mediators like α-SMA [41]. This mechanism aligns with its efficacy in IPF, where TGF-β1 hyperactivity similarly underlies disease progression [31,42].

The translational implications of these findings are significant. Current RILI management relies on glucocorticoids for acute inflammation, but lacks therapies targeting chronic fibrosis [43,44]. Nintedanib’s proven safety in IPF and systemic sclerosis-associated ILD [8,9,24] supports its repurposing for RILI, particularly in cancer patients receiving thoracic radiotherapy. However, radiation injury presents unique challenges, including combined DNA damage and immune dysregulation, which may necessitate tailored dosing regimens. Furthermore, the growing use of immunotherapy in oncology raises questions about potential synergies or antagonism with Nintedanib, given the heightened RILI risk in these patients [43]. Preclinical studies combining Nintedanib with immune checkpoint inhibitors could clarify these interactions.

Several limitations warrant consideration. First, the reliance on H&E staining (without inflammatory cell phenotyping) and total SMAD2 expression analysis (lacking phosphorylation status) may limit mechanistic precision. While widely used in fibrosis models [44,45], future studies should incorporate immunophenotyping (e.g., CD68/CD3 for macrophages/T cells) and p-SMAD2 quantification to resolve specific inflammatory subsets and TGF-β pathway activation. Second, the origin of α-SMA-positive myofibroblasts remains unresolved, as co-staining with epithelial (E-cadherin) or endothelial (CD31) markers was not performed to exclude EndMT/EMT contributions [46]. Third, the 3-month observation period and murine model constraints may not fully replicate human RILI heterogeneity. Extended follow-up and clinical validation (particularly with dose-comorbidity stratification) are needed to confirm therapeutic relevance.

## Conclusion

Our study highlights Nintedanib as a promising therapeutic strategy for RILI, capable of ameliorating both acute inflammation and chronic fibrosis through modulation of the TGF-β1/Smad2 pathway. These results not only expand the potential applications of Nintedanib but also reinforce the importance of targeting shared mechanisms in fibrotic diseases. Further investigations into optimal dosing, combination therapies, and biomarkers of treatment response will be critical for advancing this approach into clinical practice.
